# Over-Education

**Published:** 1879-10

**Authors:** 


### The Bistoury.

ELMIRA, N. Y., OCT., 1879.

The BISTOURY is published Quarterly, upon the first of April, July, October and January, at Fifty Cents a year in advance.

### OVER-EDUCATION.

The degree to which a young man should be educated is a question involving much perplexity. It has become popular to advocate higher education, so that most people strive to fill full the popular demand by keeping their children in school beyond the capacity of the child to learn or the parent to pay.

"The safety of the Republic depends upon the education of her sons," is the hackneyed expression of the stump orator. Scarcely a lecturer that does not have a flight of oratory or a peroration for the "education of the people." All this is well enough, if a decent regard is paid to the requirements and capabilities of the "people" to be educated.

Mr. Charles G. Fairman of this city, has taken a position upon this question that can not fail of approval by every thoughtful and unprejudiced mind. He claims that all young men are not capable of being classically educated, and that by far the largest number of them should be taken from their studies when they have received a "common school education," and then taught some trade, the successful following of which would afford them an independent livelihood, and the commonwealth skilled artisans.

If this statement required any defense, it is convincingly found in the fact that, not one young man in a hundred, sought to be highly educated, ever attains the mark aimed at.

We believe there is such a thing as over-educating a young man,—keeping him at the Academy or College, until he has passed the age when he should have learned something of the methods of conducting business. When he is taken from school, he finds his Latin, Greek and Geometry do not avail him in coping with the young man who parted company with him at the door of the grammar school, and who from that day, has been employed in earning a livelihood. We find too often that the young man from the college succeeds best as an employee—conducting a business engineered by the grammar school boy, who does not know so much of Virgil and the rules of rhetoric, but who has had his natural wit sharpened by knocking against those who are diligently employed in "earning a living."

We do not say that this condition of affairs obtains in every instance of a classically educated man,—by no means. The world is full of college bred gentlemen whom we could illy spare. But we do say that parents try to make scholars of children that have no capacity for such a sphere in life, and ruin their prospects by keeping them in school too long.

Just what advantage a classical education becomes to a young man whose ambition is to become a clerk in a dry goods store, and eventually himself a merchant, does not clearly appear. Aside from the pleasure it affords every gentlemen to be well informed in literature and the arts, we can not perceive that his abilities as a merchant are enhanced by spending one quarter of his life in school. Rather do we see that he has let many precious days slip by that would have brought him more knowledge of his business and better pecuniary reward.

We know this to be the unpopular side of the educational question,—for that reason few editors are found who are willing to present it. We are aware also, that we are looking at it in a business way,—estimating it according to the dollars and cents it will bring. After all, is not the monetary standard the one by which we are always most critically estimated? Is it not the one that we most ardently desire to be successfully measured by ourselves? We think so; for whatever may be the attainments of educated men, they can not be happy or wholly contented unless their personal wants are in some manner satisfied.

To accomplish this successfully and most easily, is the desire of more than ninety-nine hundredths of the people. It is for this large number we write.

We now and then encounter an over-educated man, out of employment. We count him intelligent, interesting and quite agreeable. We admire him for his qualities of mind, his politeness, and the ability with which he analyzes a flower, translates a sentence in Greek, or gives an elaborate philosophical reason why your garden pump will only throw water twenty feet when it was guaranteed to do twice as well. But, notwithstanding his varied accomplishments in letters and the sciences you do not trust him to do business for you. Now, why ?

Well, the reply generally is, " O, give me a man with less learning and more common sense ! " You say he has been kept in school too long, that he is an excellent scholar but knows nothing about business.

Then, it not infrequently happens that parents are outrageously mistaken in the mental ability of their boys, and try to make doctors or lawyers of them, keeping them at school with that view. The result is that we have too many contemptibly unsuccessful practitioners of both these professions and too few skillful artisans.

It is much easier to find fault than to suggest a remedy. But it does seem to us that too many boys are sent to college and too few taught business pursuits. We need fewer professional men and more skilled mechanics.—Fewer boys in our academies and colleges, and more in our work-shops and mines. In short, place those boys in college who show undoubted aptitude for higher education, and dismiss the others at the doors of the grammar schools.

